# The Suppressive Role of SOX7 in Hepatocarcinogenesis

**DOI:** 10.1371/journal.pone.0097433

**Published:** 2014-05-09

**Authors:** Chong Wang, Yu Guo, Jing Wang, Zhiqun Min

**Affiliations:** 1 Department of Basic Medical Sciences of Medical College, Xiamen University, Xiamen, Fujian, China; 2 Department of Hepatic Surgery, the Third Affiliated Hospital of Sun Yat-sen University, Guangzhou, Guangdong, China; 3 Department of Gynaecology and Obstetrics, the First Affiliated Hospital of Sun Yat-sen University, Guangzhou, Guangdong, China; 4 Clinical Laboratory Center of Molecular Medicine, the Second Affiliated Hospital of Guangzhou Medical University, Guangzhou, Guangdong, China; Institut für Pathologie, Greifswald, Germany, Germany

## Abstract

SOX7 is a transcription factor mediating various developmental processes. However, its role in hepatocellular carcinoma (HCC) remains unclear. Here, we assessed the role of SOX7 in hepatocarcinogenesis. We found HCC samples exhibited lower levels of SOX7 mRNA and protein expression than non-tumor samples, and the expression of SOX7 was negatively correlated with tumor size. SOX7 expression was also reduced in four HCC cell lines (SMMC-7721, Hep3B, HepG2 and Huh 7). Overexpression of SOX7 could inhibit HCC cell growth, with G1to S phase arrest. In SOX7-overexpression cells, cyclin D1 and c-myc, two cell cycle promoters, were down-regulated. Moreover, ectopic expression of cyclin D1 or c-myc could override G1 to S pahse arrest induced by SOX7. Furthermore, overexpression of SOX7 suppressed tumor formation with down-regulation of cyclin D1 and c-myc *in vivo*. The expression of Ki-67, a proliferation marker, was also reduced in SOX7-overexpression tumors. Taken together, our study suggests that SOX7 plays an important inhibitory role in hepatocarcinogenesis, and might be a novel target for HCC therapy.

## Introduction

Hepatocellular carcinoma (HCC) is one of the most common cancers in the world and has an extremely poor prognosis [1], [2]. To date, the etiology of HCC has been largely established. Chronic hepatitis B virus and hepatitis C virus infections, exposure to aflatoxin and other risk factors contribute to HCC development. However, the underlying molecular mechanisms in hepatocarcinogenesis remain unclear.

Over the past decade, extensive research has focused on the identification of cellular signaling pathways implicated in HCC. Wnt/β-catenin signaling pathway is highly conserved from nematodes to humans, and plays critical roles in both physiological and pathophysiological events [3], [4]. Wnt proteins are extracellular signaling molecules and could induce signal transduction to prevent β-catenin phosphorylation and degradation. Stabilized β-catenin then accumulates and translocates into the nucleus, and interacts primarily with T-cell factor (TCF)/lymphoid enhancer factor (LEF) high-mobility-group (HMG) box transcription factors to activate target genes. Increasing evidences have shown that aberrant activation of Wnt/β-catenin signaling is associated with the development of HCC. A high incidence of β-catenin mutations has been observed in HCC cases, resulting in accumulation of β-catenin which could contribute to HCC [5]–[7]. Hepatocytes with nuclear translocation of β-catenin display abnormal cellular proliferation [8]. Therefore, β-catenin has been regarded as a particularly attractive target in HCC therapy.

Proteins containing a HMG domain with strong amino acid similar to the HMG domain of sex-determining region Y (Sry) are grouped into SOX family. The SOX proteins function as transcription factors and bind specific DNA sequence 5′-(A/T)(A/T)CAA(A/T)G-3′ [9]. There are at least thirty SOX members and they are further divided into nine subgroups. SOX7, together with SOX17 and SOX18, belongs to SOX group F. As a transcription factor, SOX7 acts not only by activating its target genes, but also by regulating the Wnt/β-catenin pathway. SOX7 could compete with TCF/LEF activity by interaction with β-catenin and then inhibits the target genes expression [10]. It has been found that SOX7 mediates various developmental processes, including hematopoiesis, cardiogenesis, vasculogenesis, endoderm differentiation and myogenesis [11]. Recently, SOX7 is proposed to function in tumorigenesis depending on the tumor type. For example, SOX7 is up-regulated in pancreatic cancer cell lines and primary gastric cancer cases [12], but down-regulated in primary colorectal tumors, prostate cancer, lung cancer and breast cancer [13]–[16]. However, its role in HCC remains unclear.

Considering the importance of Wnt/β-catenin signaling pathway in HCC and the regulatory effects of SOX7 on this signaling, we assessed the role of SOX7 in hepatocarcinogenesis. We found the expression of SOX7 was down-regulated in HCC tissue samples and in HCC cell lines. Overexpression of SOX7 could inhibit HCC cell growth and could induce G1 to S phase arrest. The expression of cyclin D1 and c-myc, two target genes of β-catenin, was down-regulated after SOX7 restoration. Moreover, ectopic expression of cyclin D1 or c-myc could override G1 to S pahse arrest induced by SOX7. Furthermore, SOX7 could suppress tumorigenesis and cyclin D1 and c-myc expression in the xenograft mouse model. The expression of Ki-67, a proliferation marker, was also reduced in SOX7-overexpression tumors. Our study provides the evidences for the inhibitory effects of SOX7 on hepatocarcinogenesis.

## Materials and Methods

### Ethics statement

The study was approval by the Institutional Research Ethics Committees of the Third Affiliated Hospital of Sun Yat-sen university, and written informed consent was obtained from all patients. All animal procedures in this study were approved by the Animal Experimentation Ethics Committee of Lingnan Hospital, Sun Yat-sen University.

### Tissues Samples

Samples of tumor and adjacent non-tumor liver tissues were obtained from patients who had undergone primary HCC curative hepatic resection at the third affiliated hospital of Sun Yat-sen university, Guangzhou, China. Immediately after resection, all tissues were snap-frozen in liquid nitrogen and stored at −80°C.

### Cell lines, Constructs and Transfection

Human hepatocyte cells (L02) and HCC cell lines (SMMC-7721, Hep3B, HepG2 and Huh 7) were cultured as reported [17]. The human SOX7, cyclin D1 and c-myc genes were cloned from L02 cells using the following primers: SOX7, forward, 5′-ATGGCTTCGCTGCTGGGAG-3′, reverse, 5′-CGCCTCCAGCTCTATGACACAC-3′; cyclin D1, forward, 5′-ATGGAACACCAGCTCCTGTG-3′, reverse, 5′-TCAGATGTCCACGTCCCG-3′; c-myc, forward, 5′-CTGGATTTTTTTCGGGTAGTG-3′, reverse, 5′-TTACGCACAAGAGTTCCGTAG-3′. The resulting PCR products were inserted into pcDNA3.1(-) expression vector (Invitrogen) to obtain SOX7, cyclin D1 and c-myc expression vector respectively. The hairpin small interfering RNA (siRNA) was used to knockdown SOX7. The target sequence for SOX7 is: siSOX7a, 5′-acgccgagctgtcggatgg-3′, and siSOX7b, 5′-ggaatgttcactgacgtct-3′. A random sequence was used as a negative control (NC): 5′-gtgcgctgctggtgccaac-3′. The siRNAs were inserted into pSilence 4.1-CMV-neo vector (Ambion Inc.) to generate siSOX7a, siSOX7b and siNC plasmids. All transfections used Lipofectamine 2000 (Invitrogen) according to the manufacturer′s protocol. G418 (500 µg/ml, Sigma-Aldrich) was used to select the transfected cells.

### Immunohistochemistry

Immunohistochemistry analysis was performed as described previously [17]. Briefly, the slides were deparaffinized, and endogenous peroxidase activity was quenched by hydrogen peroxidase. Antigen retrieval was performed by microwave-heating in sodium citrate buffer. Sections were blocked with 3% normal serum and then incubated with anti-SOX7 antibody (Santa Cruz, sc-20093, 1∶500). Bound antibody was detected by the avidin-biotin-peroxidase complex method, using the Elite ABC kit (Vector Laboratories) as recommended by the manufacturer. All the experiments were performed independently three times at least.

### Western Blotting

Cells or tissues lysates were separated by SDS-poly-acrylamide gel electrophoresis and transferred to poly-vinylidene difluoride membranes (Amersham). Membranes were blocked with 5% non-fat milk and then probed with antibodies against SOX7 (Santa Cruz, sc-20093, 1∶1000), cyclin D1 (Santa Cruz, sc-246, 1∶1000), c-myc (Santa Cruz, sc-40, 1∶1000) and GAPDH (Santa Cruz, sc-32233, 1∶2000) as indicated. After washing, the membranes were incubated with horseradish peroxidase-conjugated secondary antibodies and visualized using the ECL system (Amersham). All the experiments were performed independently three times at least.

### Colony Formation Assay

Hep3B and SMMC-7721 cells were transfected with either empty vector or SOX7 expression vector. Cells were plated on 6-well culture dishes, and G418 (500 µg/ml, Sigma-Aldrich) was added to the culture media to select the transfected cells. Every 3 days the medium was replaced with fresh medium containing G418. Two weeks later, colonies were stained by crystal violet and counted. All the experiments were performed three times at least.

### MTT Viability Assay

The viability of the cells was assessed by 3-(4,5-dimethylthiazol-2-yl)-2,5-diphenyltetrazolium bromide (MTT, Sigma-Aldrich) assay. A total of 1×10^3^ cells per well were plated in 96-well with triplicate wells for each transfection, and incubated for 24 h in 100 µl culture media. Cells were transfected with either vector or SOX7. MTT (500 mg/ml) was added to the cells and cultivated for another 4 h. After the medium was aspirated, the cells were dissolved by dimethyl sulfoxide (Sigma-Aldrich). Absorbance of the formazan product was measured by an enzyme-linked immunosorbent assay reader. Each assay was repeated three times.

### Flow Cytometric Analysis

The effect of SOX7 on cell cycle was checked in Hep3B or SMMC-7721 cells by propidium iodide staining and flow cytometry. Briefly, 1×10^6^ cells were harvested at 48 h after transfection, washed in PBS and fixed in ice cold 70% ethanol for 1 hour. RNA was digested by incubating the samples with 1 mg/ml RNase A (Invitrogen) for 30 min at 37°C. Propidium iodide (50 µg/ml, Sigma-Aldrich) was then added and the samples were recorded using the Navios Flow Cytometers (Beckman Coulter). Cell cycle analysis was performed with the use of Multi Cycle for Windows (Phoenix Flow Systems). Experiments were repeated in triplicate. Average values and standard deviation statistical analyses were computed.

### *In Vivo* Tumorigenesis Assay

Hep3B or SMMC-7721cells transfected with vector or SOX7 were injected subcutaneously into the dorsal left flank of 4-week-old male Balb/c nude mice. Six mice were used in each group. Tumor diameter was measured every 3 days for 4 weeks. Tumor volume (mm^3^) was calculated using the following formula: volume  = 0.5× (shortest diameter)^2^×(longest diameter). Four weeks later, mice were sacrificed and the tumor weights were measured.

### Statistical Analysis

Data were presented as the mean ± SD error of the mean. Student's t test was used for comparison among different groups. The correlation of SOX7 expression with various clinicopathologic parameters were calculated with χ^2^ test. The difference in tumor growth rate between the two groups of nude mice was determined by repeated-measures analysis of variance. *p*<0.05 was considered statistically significant.

## Results

### SOX7 was down-regulated in HCC tissues and in HCC cell lines

**Figure 1:**
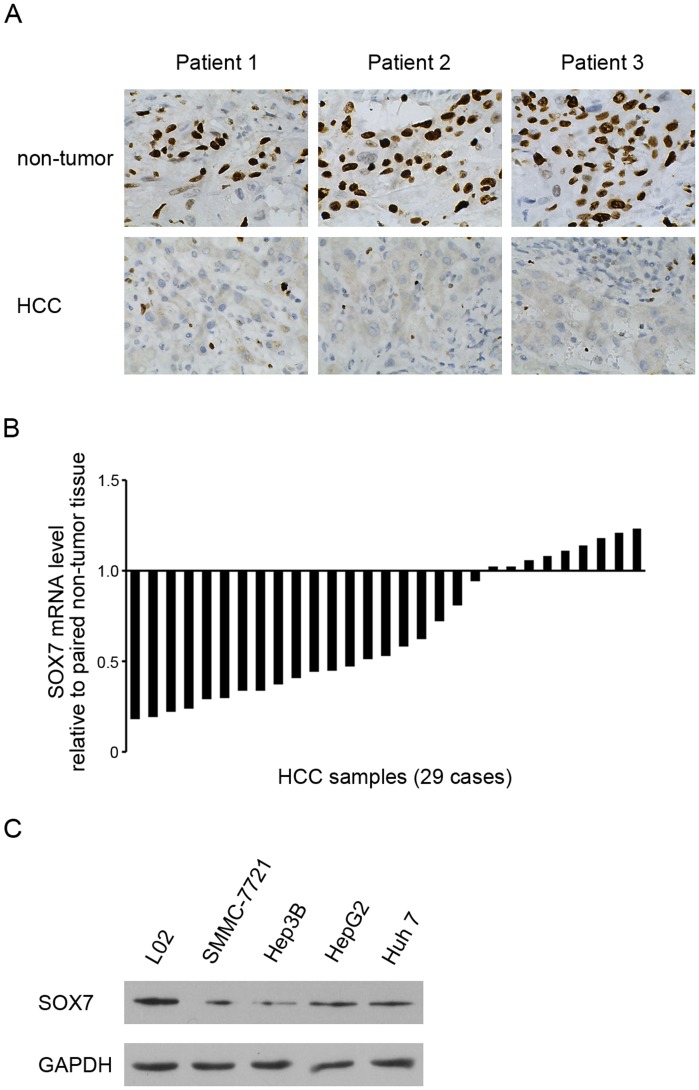
SOX7 was down-regulated in HCC tissues and in HCC cell lines. (A) Immunohistochemistry analysis of SOX7 expression in HCC and adjacent non-tumor liver samples. Representative photographs from three patients. (B) RT-PCR analysis of SOX7 mRNA expression in paired HCC samples. (C) Western Blotting analysis of SOX7 expression in HCC cell lines (SMMC-7721, Hep3B, HepG2 and Huh 7) and normal hepatocyte cell line (L02). GAPDH expression levels were used as internal controls. * indicates *p*<0.05. The experiments were performed independently three times at least.

To study the role of SOX7 in HCC, we first examined the expression pattern of SOX7 in 29 paired HCC samples and adjacent non-tumor tissue samples by immunohistochemistry. Compared with non-tumor samples, HCC samples exhibited lower levels of SOX7 protein expression (Fig. 1A). The expression of SOX7 was negatively correlated with tumor size (Table 1). Quantitative RT-PCR analysis showed that SOX7 mRNA was frequently decreased in HCC samples (Fig. 1B). Next we detected SOX7 expression in HCC cell lines (SMMC-7721, Hep3B, HepG2 and Huh 7) and normal hepatocyte cell line (L02). SOX7 was reduced in all the HCC cell lines, compared with that in L02 cells (Fig. 1C). These data demonstrated that SOX7 was down-regulated in HCC tissue and in HCC cell lines.

**Table 1:** Relationship between SOX7 expression and clinicopathologic features of patients with hepatocellular carcinoma.

Features	High SOX7 expression	Low SOX7 expression	p value
**Mean age (years)**	61.8	63.2	0.47
**Gender**			0.82
Male	5	12	
Female	4	8	
**Tumor Size (cm)**			0.03
<2	7	7	
≥2	2	13	
**Differentiation**			0.12
Well	5	5	
Moderate	3	5	
Poor	1	10	
**Liver cirrhosis**			0.06
Yes	6	6	
No	3	14	
**Metastasis**			0.64
Yes	3	5	
No	6	15	
**HBsAg status**			0.60
Positive	4	11	
Negative	5	9	
**Serum AFP**			0.06
Positive	3	14	
Negative	6	6	

### SOX7 overexpression inhibits HCC cell growth

**Figure 2:**
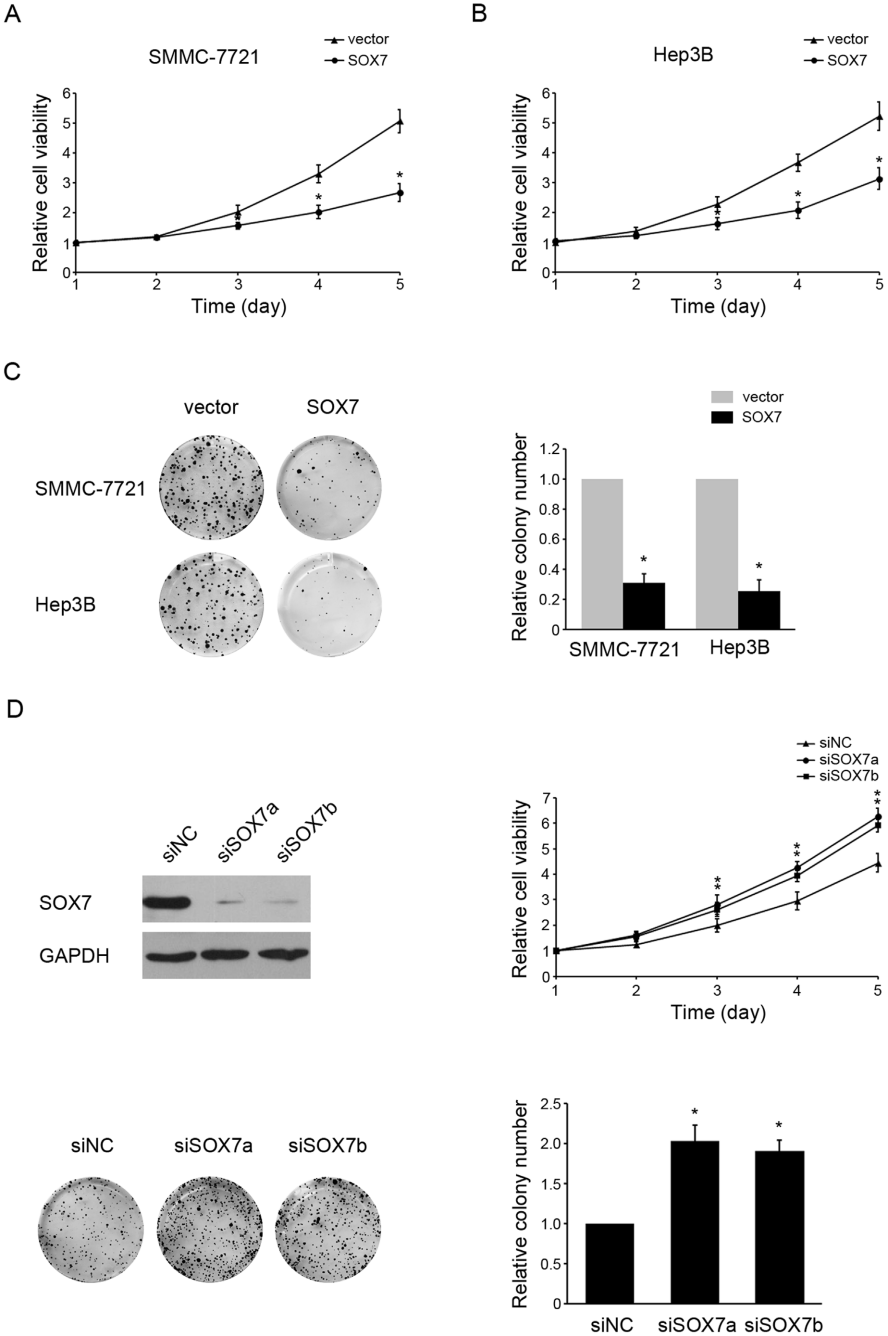
SOX7 overexpression inhibited HCC cell growth. Overexpression of SOX7 inhibited cell growth as determined by MTT assay and colony formation assay. Viability of SOX7-transfected or vector-transfected SMMC-7721(A) and Hep3B (B) cells were determined on days 1 to 5. (C) Representative pictures (left panel) and quantification (right panel) of crystal violet-stained SOX7-transfected or vector-transfected SMMC-7721 and Hep3B cells. (D) Both SOX7-specific siRNAs (siSOX7a and siSOX7b) silenced SOX7 in L02 cells successfully. SOX7 expression was detected by western blotting. (E) Viability of L02 cells transfected with siRNAs (siSOX7a and siSOX7b) or siNC were determined on days 1 to 5. (F) Representative pictures (left panel) and quantification (right panel) of crystal violet-stained L02 cells transfected with siRNAs (siSOX7a and siSOX7b) or siNC. Each bar represents the average ± SD of three independent experiments. * indicates *p*<0.05.

To address the function of SOX7 down-regulation in HCC, we examined the effects of SOX7 on cell growth by MTT assay and colony formation assay. Hep3B and SMMC-7721 cells were transfected with either empty vector or SOX7 expression vector. Reduction of viability was observed in SOX7-overexpressed cells compared with cells transfected with empty vector (Fig. 2A, 2B). Moreover, SOX7-overexpressed cells formed fewer colonies than the control cells (Fig. 2C). Similar results were observed in both two types of HCC cells. Because of low expression of SOX7 in HCC cells, we examined the effects of SOX7 knockdown on cell growth in nontumorigenic L02 cells. It was confirmed that SOX7 was knockdown effectively by both siRNAs (Fig. 2D). Both MTT assay and colony formation assay indicated that SOX7 knockdown promoted proliferation of L02 cells (Fig. 2E, 2F). Collectively, these data suggested that SOX7 had an anti-proliferation roleHCC.

### SOX7 overexpression induces G1 to S phase arrest of HCC cells by down-regulation of cyclin D1 and c-myc

**Figure 3:**
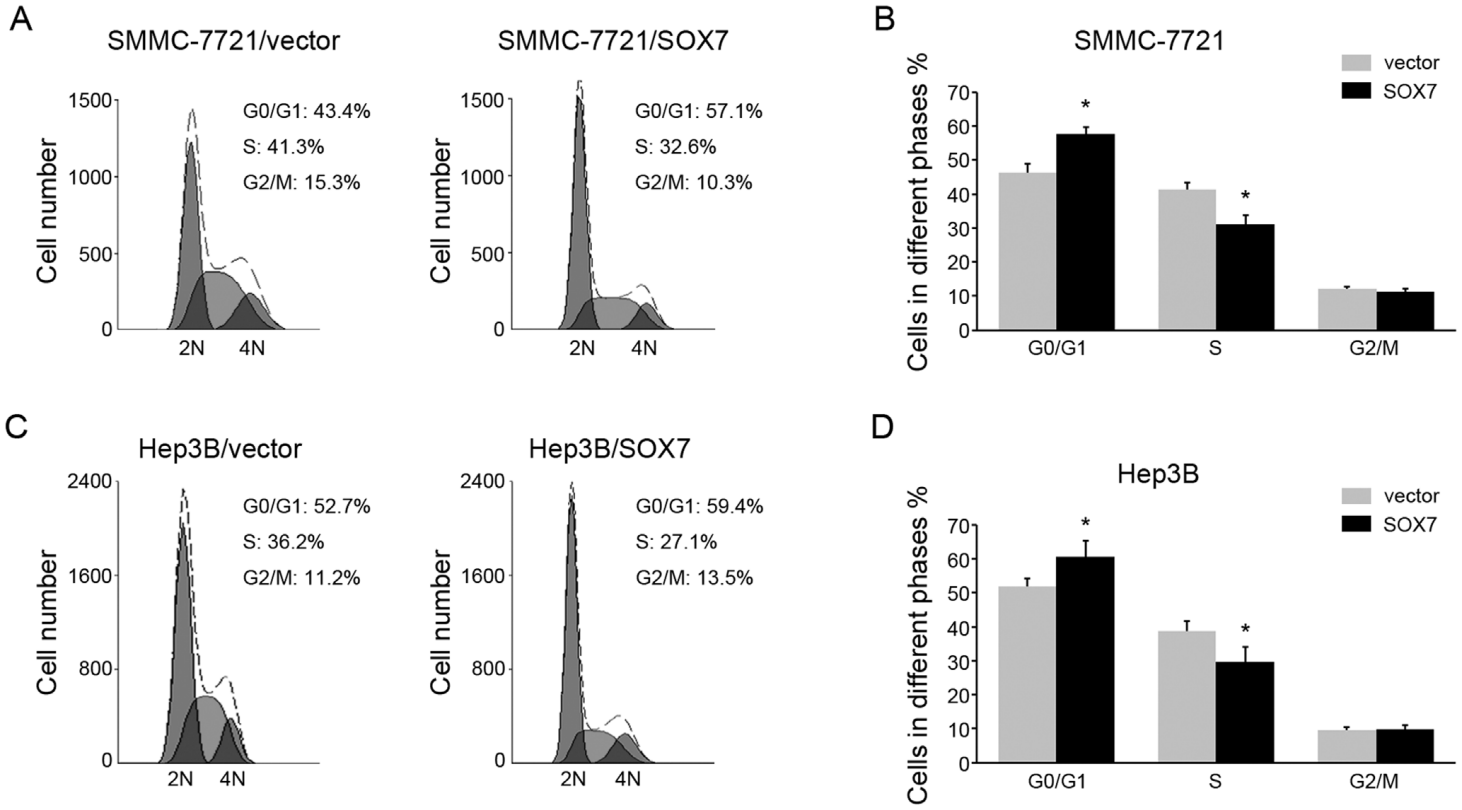
Overexpression of SOX7 inhibited cell cycle. (A) Representative pictures of flow cytometric analysis of SMMC-7721cells transfected with vector or SOX7. (B) The cell percentages in G0/G1, S and G2/M phase were measured. (C) Representative pictures of flow cytometric analysis of Hep3B cells transfected with vector or SOX7. (D) The cell percentages in G0/G1, S and G2/M phase were measured. * indicates *p*<0.05. The experiments were performed independently three times at least.

**Figure 4:**
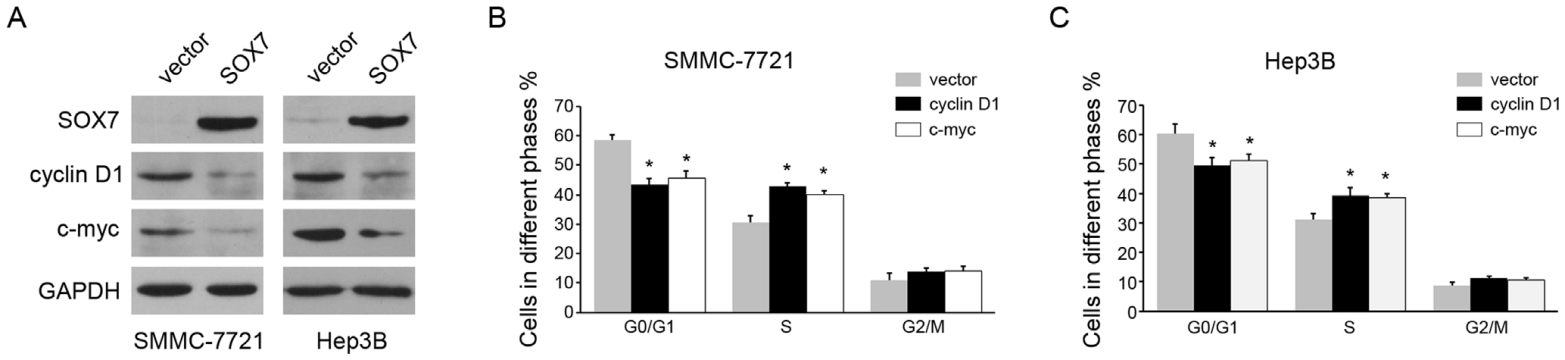
SOX7 induced G1 to S phase arrest by down-regulation of cyclin D1 and c-myc. (A) Western blotting analysis of SOX7, cyclin D1, c-myc and GAPDH levels in SMMC-7721 and Hep3B cells transfected with vector or SOX7. (B, C) Overexpression of cyclin D1 and c-myc could override G1 to S pahse arrest induced by SOX7. Cyclin D1 or c-myc was overexpressed in SOX7-overexpression cells, and the cell percentages in G0/G1, S and G2/M phase were measured by flow cytometric analysis. * indicates *p*<0.05. The experiments were performed independently three times at least.

Since SOX7 could inhibit HCC cell growth, we further investigated the mechanism that mediated the anti-proliferation function. Flow cytometry was performed to examine the effects of SOX7 on cell cycle. We found overexpression of SOX7 increased the percentage of cells in G0/G1 peak but decreased that in S peak (Fig. 3), indicating that SOX7 could induce G1 to S phase arrest. Accordingly, we tested the expression of a panel of proteins involved in cell cycle. SOX7 overexpression had no effect on the expression of CDK4, and CDK6. However, the expression of two cell cycle promoters, cyclin D1 and c-myc, were down-regulated (Fig. 4A). Furthermore, to determine if cyclin D1 and c-my function downstream of SOX7, we overexpressed ectopic cyclin D1 or c-myc in SOX7-overexpression cells and examined the cell cycle. It is found that either cyclin D1 or c-myc overexpression could override the G1 to S pahse arrest induced by SOX7 (Fig. 4B, 4C). These data suggested SOX7 induced G1 to S phase arrest by down-regulation of cyclin D1 and c-myc.

### Overexpression of SOX7 Suppresses Tumor Formation

**Figure 5:**
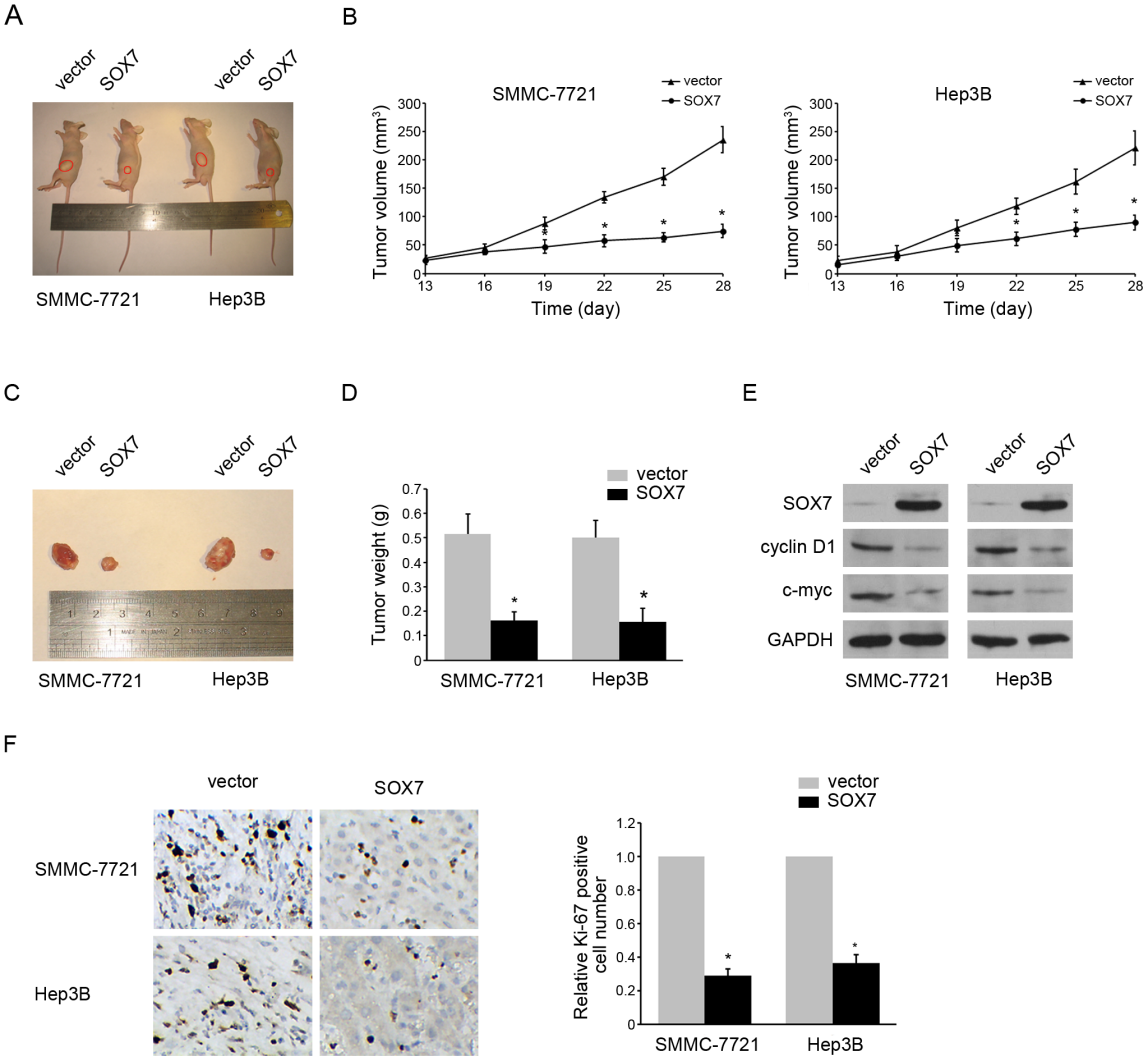
Overexpression of SOX7 suppressed tumor formation. (A) A representative picture of tumor growth in nude mice subcutaneously inoculated with vector or SOX7 transfected HCC cells. The tumors were outlined by red circle. (B) Subcutaneous tumor growth curve of nude mice with different treatment. (C) A representative picture of the isolated tumors. (D) The mean tumor weights in nude mice with different treatment. (E) Western blotting analysis of SOX7, cyclin D1, c-myc and GAPDH levels in the subcutaneous tumor samples. (F) Representative pictures (left panel) and quantification (right panel) of immunohistochemistry-detected Ki-67 expression in the subcutaneous tumor samples. The data were means ± SD of three separate experiments. * indicates *p*<0.05.

To investigate the effects of SOX7 on tumorgenesis *in vivo*, HCC cells transfected with vector or SOX7 were injected subcutaneously into nude mice to initiate tumor formation. Four weeks later, large tumors were seen in the vector groups, while the tumor volume was still minimal in those mice transplanted with the SOX7-expression cells (Fig. 5A, 5B). At the end of experiments tumors were isolated (Fig. 5C) and weighed. Tumors from SOX7-transfected nude mice weighed significantly less than the control mice (Fig. 5D). These results were consistent with the anti-proliferation function of SOX7 and indicated that SOX7 overexpression elicited a strong anti-tumor effect on HCC *in vivo*. We also analyzed the cyclin D1 and c-myc expression in these tumors. SOX7 was overexpressed successfully, and the expression of cyclin D1 and c-myc was reduced in SOX7-overexpression tumors (Fig. 5E), similar to the results *in vitro*. To confirm the anti-proliferation role of SOX7 *in vivo*, we detected the expression of Ki-67, a proliferation marker, in above tumors. Ki-67 expression was significantly decreased after SOX7 overexpression (Fig. 5F), indicating that SOX7 could inhibit proliferation *in vivo*. Taken together, we found that overexpression of SOX7 suppressed tumor formation by proliferation inhibition *via* down-regulation of cyclin D1 and c-myc.

## Discussion

SOX7 has been regarded as a key regulator in embryonic development. Recently, some studies show that SOX7 plays an important role in tumorigenesis. Indeed, SOX7 is up-regulated in pancreatic cancer cell lines and primary gastric cancer cases, but down-regulated in primary colorectal tumors, prostate cancer, lung cancer and breast cancer, indicating that the role of SOX7 in tumorigenesis is depending on tumor type. However, the role of SOX7 in HCC remains unclear. The SOX7 gene is located on the short arm of chromosome 8 [10]. Interesting, HCC frequently shows loss of heterozygosity at loci on this region [18], [19], indicating a potential role of SOX7 deletion in HCC. In this study, we showed that both SOX7 mRNA and protein expression were frequently decreased in HCC tissues, and the expression of SOX7 was negatively correlated with tumor size. These data raised the possibility of the reduction of SOX7 expression as a detection marker for HCC.

SOX7 has been proposed to function as a tumor suppressor in some cancers. Similarly, our functional studies suggested that SOX7 could inhibit HCC cell growth both *in vitro* and *in vivo*. The results were consistent with another study published recently, which showed SOX7 knockdown promoted proliferation of HCC cells [20]. These data indicated that SOX7 was sufficient and necessary for HCC cell growth inhibition. The inhibitory effects might result from change of β-catenin signaling pathway. SOX7 could interact with β-catenin to compete with TCF/LEF activity, and then inhibits target genes expression [10]. In cancer cell lines, overexpression of SOX7 blocks β-catenin-mediated transcriptional activity. In HCC cells, we found SOX7 could reduce the expression of c-myc and cyclin D1, two target genes of β-catenin. The two proteins control the cell cycle and play an important role in HCC [21], [22]. Consistently, SOX7 overexpression could inhibit cell cycle *via* inducing G1 to S phase arrest. Moreover, the cell cycle arrest could be overrided by ectopic expression of cyclin D1 and c-myc. We also found SOX7 down-regulated c-myc and cyclin D1 *in vivo*. Therefore, we concluded that SOX7 down-regulated c-myc and cyclin D1 to inhibit cell cycle and HCC cell growth.

In summary, we identified that SOX7 could suppress tumor growth in HCC, and our investigation suggested that restoration of SOX7 would be a potential molecular target for HCC therapy.
